# Chronic Calcaneovalgus Deformity Treated With Tibialis Anterior Tendon Transversion: A Case Report

**DOI:** 10.7759/cureus.30576

**Published:** 2022-10-22

**Authors:** Omar A Batouk, Turki H Alhassani, Eyad J Algahwaji

**Affiliations:** 1 Orthopedic Surgery, King Saud bin Abdulaziz University for Health Sciences, College of Medicine, Jeddah, SAU; 2 Medicine, Umm Al-Qura University, Makkah, SAU

**Keywords:** spina bifida, calcaneus, calcaneovalgus, foot deformities, meningomyelocele

## Abstract

We present a 21-year-old female with a previously known myelomeningocele who underwent myelomeningocele repair 10 years ago. She presented to the orthopedic outpatient clinic with bilateral calcaneovalgus deformity, causing non-healing ulcers and multiple hospitalizations for pressure ulcers, cellulitis, and osteomyelitis. She had successful tibialis anterior transfer surgery on her right foot six years ago. The patient arrived for treatment of her left foot deformity to underwent three hours of surgery that was uneventful without any complications and recovered well postoperatively and was discharged on day 2. On week 1, the patient came to the clinic for follow-up; the wound was healthy, placed in the full cast in the equinus position, and referred to physiotherapy. In the third month postoperatively, she was able to tolerate her weight with her foot back to a neutral position with full dorsiflexion.

## Introduction

Spina bifida is neural element myelodysplasia that presents in the vertebrae as a deformity in the posterior components. Spinal cord and nerve root dysplasia cause bowel, bladder, motor, and sensory paralysis below the lesion level. Spina bifida patients frequently have other associated spinal cord injuries or structural brain abnormalities that might impair neurological function [1]. Myelomeningocele is more common in the lumbar area. It is related to orthopedic deformities such as calcaneovalgus, which occurs when the foot’s lateral extensors and ankle evertors overwhelm the anterior and posterior tibialis [2]. Calcaneovalgus abnormalities cause significant weight-bearing on the heels, resulting in pressure ulcers, skin infections, or even osteomyelitis. Early surgical correction is advised to avoid deformity development and stiffness. Passive stretching of the restricted dorsiflexion in infants should begin as soon as feasible and can be assisted with correction splints [3]. In adults, the surgical intervention aims to achieve plantigrade, painless foot stabilization that can be braced and avoid subsequent osseous abnormalities. In this case, we present a 21-year-old female patient who came to our outpatient orthopedic clinic with bilateral calcaneovalgus deformity contributing to multiple hospital admissions due to heel pressure ulcers, cellulitis, and osteomyelitis that made her decide to precede with bilateral tibialis anterior tendon transversion. After three months of surgery, the patient tolerated her weight, her foot was back to its neutral position, and she maintain her daily life activities.

## Case presentation

**Figure 1:**
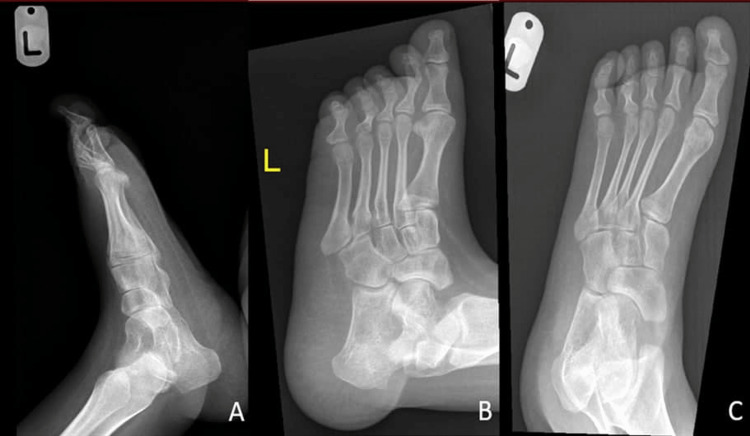
Preoperative X-ray of the left foot: (A) lateral, (B) oblique, and (C) anteroposterior views

**Figure 2:**
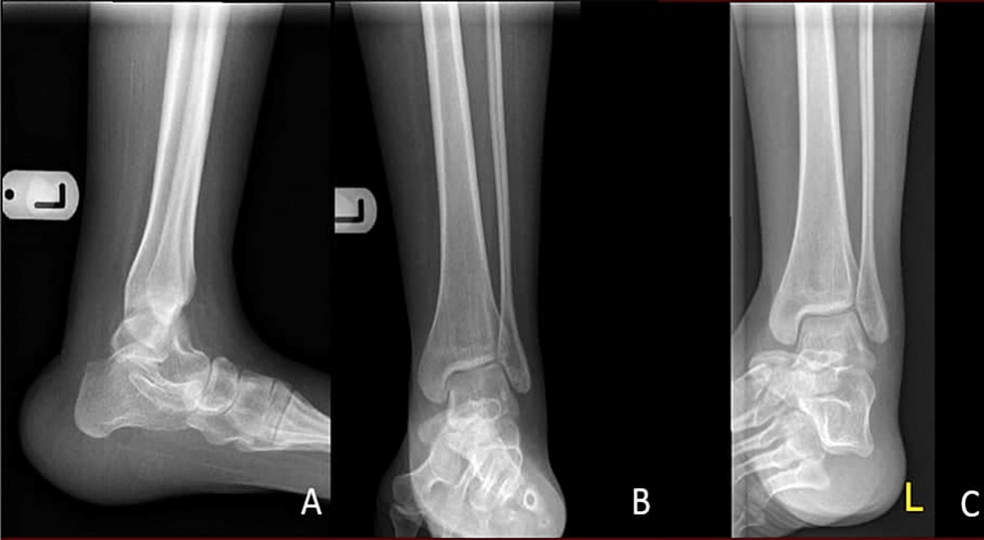
Preoperative X-ray of the left ankle: (A) lateral, (B) anteroposterior, and (C) mortise views

A 21-year-old female, a previously known case of myelomeningocele at lumber regions, underwent myelomeningocele repair 10 years ago. She presented to the orthopedic outpatient clinic with bilateral calcaneovalgus deformity causing non-healing ulcers and several admissions to the hospital due to heel pressure ulcers, cellulitis, and osteomyelitis (Figure 1 and Figure 2).

She underwent tibialis anterior transfer surgery six years ago on her right foot with the healing of the heel pressure ulcer and was able to voluntary plantarflexed the foot after correction of the calcaneus deformity. Now, the patient is present for correction of her left foot deformity. On examination, rigid calcaneovalgus deformity on the left foot and ankle was 45 degrees dorsiflexed with callosities over the heel and subluxation of metatarsal joints.

**Figure 3:**
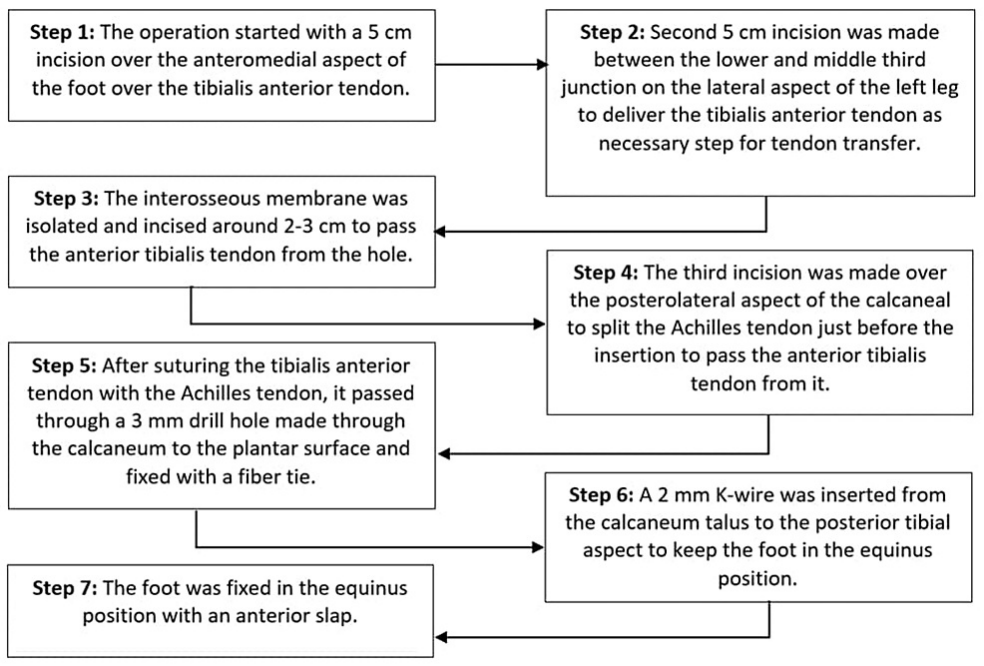
Flowchart presenting the steps of the surgery

The treatment plan has been discussed with the patient. As a result, the doctors decided to reconstruct the left foot using the tibialis anterior tendon transversion, as this aim to maintain independent ambulation and limit hospital admission from infections and pressure ulcers as the patient has a good history with the previous reconstruction of her right foot that minimized her hospital admissions. Therefore, the decision was made to proceed with surgical reconstruction of the left foot with tibialis anterior tendon transversion. The surgery steps are mentioned in Figure 3.

**Figure 4:**
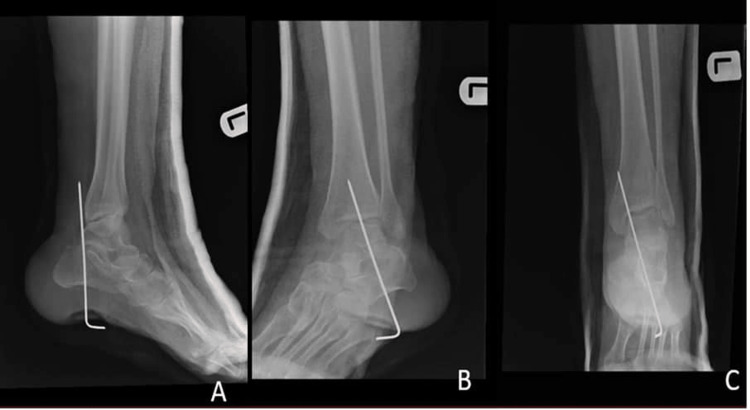
Postoperative X-ray of the left ankle: (A) lateral, (B) oblique, and (C) anteroposterior views

The surgery took about two hours, and the patient recovered well postoperatively without any complications and was discharged on day 2. On week 1, the patient came to the clinic for follow-up; the wound was healthy, placed in the full cast in the equinus position, and referred to physiotherapy. In the sixth week, the cast and K-wire were removed (Figure 4).

**Figure 5:**
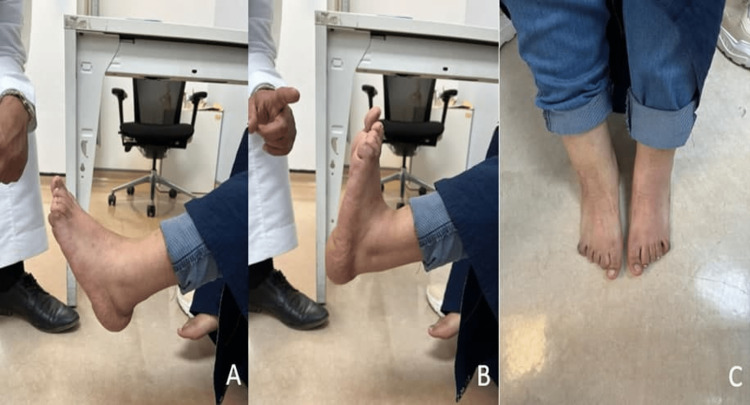
Postoperative pictures of the left ankle and foot: (A) plantar flexion, (B) dorsiflexion, and (C) picture of the left and right foot in plantigrade

**Figure 6:**
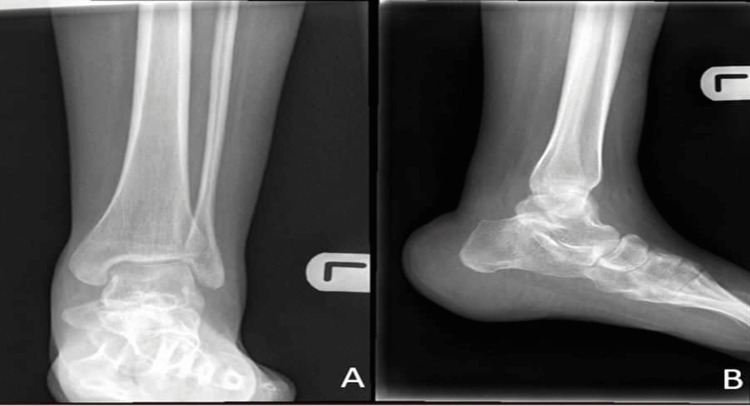
Three months after surgery and removal of K-wire: (A) anteroposterior and (B) lateral views

After three months, the patient can tolerate her weight, and her foot was back to its neutral position with the ability to fully dorsiflex the foot and limited plantarflexion. Regarding the patient’s daily activity, she is able to do her daily activity without any restrictions (Figure 5 and Figure 6).

## Discussion

In developed countries, untreated and chronic severe calcaneovalgus foot abnormalities due to spina bifida rarely remain until maturity. A calcaneovalgus is an uncommon myelomeningocele deformity that accounts for 5%-10% of all foot defects [3]. The muscles are balanced without weight-bearing, but they lack the strength to maintain the typical architecture of the foot in stance or during walking. The forefoot deviates laterally as the talus subluxated from the calcaneus. The foot’s medial arch flattens, leading to callosities, and ulceration forms along the medial border. Chronic calcaneovalgus abnormalities in adults are difficult to manage and rebuild because they typically develop in stiffness and secondary osseous deformities such as external tibial torsion. Pressure ulcers typically form over the heels in the fraction of patients who continue ambulant [2]. The treatment should be customized to each patient’s specific needs, although even in non-ambulant individuals, reconstruction may be advantageous in allowing footwear and facilitating wheelchair placement. A plantigrade, stable, and brace-able foot should be the goal of surgical reconstruction [4].

After reviewing the literature, there is no single best treatment option for calcaneovalgus in adult patients. There are many articles that give the experience of using a single approach in a specific group of patients. Park et al. looked at 31 patients with tibialis anterior tendon transfer for an average of 47 months. The results indicated that none of the patients had a recurrence or worsening of the deformity, and no other forms of foot deformity emerged following surgery. In the stance phase of gait, postoperative kinematic investigations revealed a large increase in peak plantar flexion and a significant decrease in the peak dorsiflexion force of the ankle. In addition, peak pressures beneath the forefoot and midfoot improved following surgery, and the ratio of weight-bearing on the heel to the forefoot moved toward equal weight-bearing [5]. On the other hand, a study done by Kelley et al. described the results of 28 individuals with spina bifida who received 43 Ilizarov lower extremity deformity corrections. In the 43 surgeries, there were 12 problems, five obstacles, and 13 complications. In seven individuals, further surgeries were required [6]. Bliss and Menelaus studied 25 cases of mature individuals. All patients had been observed for at least 12 years. Forty-six transfers had been done. At the follow-up, four of the 46 transferred muscles were working in a plantigrade foot, 11 were working although the foot still had a calcaneal defect, and 17 transferred muscles had never worked [7]. Rodrigues and Dias examined 76 feet that had been surgically treated for calcaneovalgus deformity by simple anterior release or anterolateral release. The results were evaluated in consideration of bracing and shoe wear issues, as well as the existence of pressure ulcers. The average follow-up duration was 4.66 years, with 62 feet yielding a favorable result and 14 yielding a poor result [8]. This patient underwent tibialis anterior transfer as this simple procedure requires only three incisions and weekly follow-up in the clinic in addition to the physiotherapy referral; as a result, a six-week cast was removed, and the three-month K-wire was removed.

The patient could tolerate her weight and foot back to its neutral position with the ability to fully dorsiflex her foot with limited plantarflexion. From the literature, the Ilizarov technique has been used to treat bilateral calcaneovalgus deformity with gradual correction over four months daily by the patient with close weekly follow-ups in the clinic. After four months, postoperatively, the foot was back to a neutral position, and the frame was removed. A below-knee cast was applied after the removal of the frame, and full weight-bearing as tolerated was allowed. Casting was kept for six weeks before the removal, and she was fitted with an ankle-foot orthosis. On review in the clinic four weeks subsequently, the patient was pain-free, with a stable, plantigrade foot [4].

## Conclusions

In conclusion, almost all myelomeningocele patients will have foot abnormalities, including calcaneus, equinus, varus, valgus, or a combination of these deformities. Foot abnormalities not only impair the visual look of the foot but also cause ambulation issues, making bracing and shoe usage challenging. In addition, skin irritation caused by foot deformities might lead to breakdown and pressure ulcers. In a patient with ambulation potential, tibialis anterior tendon transfer provides an effective and quick result compared to the Ilizarov technique, which provides gradual correction and requires daily patient adjustments over a long period, which may be difficult for the patient to adhere to. The goal of management for spina bifida foot deformity is to achieve a plantigrade, pain-free, and brace-able foot and motorize and functionalize the plantarflexion to keep the pared on independent while doing gait with good balance and push-off.
